# Differences Between Health Workers and General Population in Risk Perception, Behaviors, and Psychological Distress Related to COVID-19 Spread in Italy

**DOI:** 10.3389/fpsyg.2020.02166

**Published:** 2020-09-04

**Authors:** Luca Simione, Camilla Gnagnarella

**Affiliations:** ^1^Istituto di Scienze e Tecnologie della Cognizione, CNR, Rome, Italy; ^2^Department of Neurology and Psychiatry, Sapienza University of Rome, Rome, Italy

**Keywords:** healthcare workers, risk perception, worry, COVID-19, coronavirus outbreak, distress, mental health, SARS-CoV-2

## Abstract

In this study, we investigated the perception of risk and the worries about COVID-19 infection in both healthcare workers and the general population in Italy. We studied the difference in risk perception in these two groups and how this related to demographic variables and psychological factors such as stress, anxiety, and death anxiety. To this aim, we administered an online questionnaire about COVID-19 together with other questionnaires assessing the psychological condition of participants. First, we found that the exposition to infection risk, due to living area or job, increased the perceived stress and anxiety (i.e., medical staff in North Italy was more stressed and anxious with respect to both medical- and non-medical participants from Center and South Italy). Then, we conducted hierarchical logistic regression models on our data to assess the response odds ratio relatively to each regressor on each dependent variable. We found that health workers reported higher risk perception, level of worry, and knowledge as related to COVID-19 infection compared to the general population. Psychological state, sex, and living area were less related to these factors. Instead, judgments about behaviors and containment rules were more linked to demographics, such as sex. We discussed these results in the light of risk factors for psychological distress and possible interventions to meet the psychological needs of healthcare workers.

## Introduction

On December 31, 2019, some cases of pneumonia of unknown etiology have emerged in the Hubei region of China. Then, on January 07, 2020, the causative agent has been identified by means of oropharyngeal swabs, i.e., a virus belonging to the Coronaviridae family called SARS-CoV-2 (severe acute respiratory syndrome coronavirus 2). This new coronavirus was responsible of the respiratory syndrome called COVID-19 (World Health Organization [WHO], 2020). Most patients with positive swab test developed only minor symptoms, such as fever, dry cough, and pharyngitis, with a benign evolution and spontaneous resolution of the clinical picture. However, some patients developed severe complications, such as interstitial pneumoniae with acute respiratory distress syndrome, pulmonary edema, multiorgan failure, septic shock, and even death (Sohrabi et al., 2020). Patients at risk were especially males, aged older than 60 years, suffering from cardiovascular comorbidities (e.g., arterial hypertension, diabetes, and chronic coronary artery disease), and affected by chronic pneumopathies or cancer (World Health Organization [WHO], 2020; Zhou et al., 2020).

In the following month, the disease spread to other countries outside China, including Italy, where the first positive cases were found on February 21, 2020 (Spina et al., 2020). Italy experienced a significant increase in new cases, mostly in the month of March, in particular in the North regions, and this caused in turn a growing alarm throughout the Italian medical-hospital sector due to the imbalance between the resources of the national health system (indicated as SSN, i.e., “Sistema Sanitario Nazionale”) and the expected need for treatment required by forecasts on the virus spread. This concern was publicly expressed in the guidelines published by the Italian Society of Anesthesia, Analgesia, and Intensive Care (named SIAARTI, i.e., “Società Italiana Anestesia, Analgesia, Rianimazione e Terapia Intensiva”) on March 06, 2020, which reported that in case of huge imbalance between the population clinical needs and the effective availability of intensive resources, medical doctors should have selected patients for intensive therapies based on their actual hopes of survival (SIAARTI, 2020). In fact, in Italy, there were about 5,200 beds in total for intensive care units, and on March 11, 2020, 1,028 of these beds had already been destined to patients suffering from COVID-19. According to the predicted number of new cases, the peak of contagions would have been reached by mid of April, when at least 4,000 beds in the intensive care units would have been needed in order to treat patients with COVID-19 (Remuzzi and Remuzzi, 2020), with significant consequences also for patients not affected by COVID-19, who would have given less assistance in the aforementioned units.

However, on the one hand, doctors and other health workers multiplied their alarms relatively to this critical situation and to the related recommendations regarding behaviors to be followed and the hygienic conduct to be implemented; on the other hand, there were daily episodes of violation of such medical recommendations by the population, apparently only scarcely aware of the problem. For this reason, i.e., the failure of the unanimous spontaneous compliance of the population to the proposed hygienic rules and health practices, since February 23 the Italian Government implemented increasingly restrictive dispositions to limit the spread of the disease throughout the country with various Prime Minister Decrees (named DPCM, i.e., Decreto del Presidente del Consiglio dei Ministri; see DPCM on February 23, March 01, March 04, March 08, March 09, and March 11, 2020). In fact, a significant portion of population continued to engage in risky behaviors, prompting increasingly stricter rules emanated by the authorities. Therefore, a gap appeared to emerge between the indications and requests from the national health system staff and the reception of these same indications by the population, as well as a general difference in the perception and evaluation of the risks associated with the COVID-19 infection between the two groups. Such a difference seemed more evident especially in the areas of central and southern Italy, where the COVID-19 spread was lower than those of northern Italy, as reported by the daily data provided by the national civil protection (see Cereda et al., 2020).

The spread of the SARS-CoV virus in 2002 has shown how this type of epidemic disease has important psychopathological consequences, in the short and long term, in particular on health workers (Sim and Chua, 2004; Lung et al., 2009; Maunder, 2009). Thus, in the actual spread of the new SARS-CoV-2 virus attention to psychological health of doctors and others healthcare workers had already been expressed regarding the Chinese situation relating to COVID-19 (see for example Xiang et al., 2020), with proposals for intervention and support from the hospital structures (Chen et al., 2020). In fact, Chinese health workers in Wuhan faced a situation characterized by poor safety and protection, with excessive workloads, high infectious risk, absence of adequate personal protective equipment, and shortage of staff. This risky situation for one’s own and loved ones’ health could have clinical consequences, but also psychic ones. In fact, these health workers showed a symptomatology characterized by tiredness, worry, fear, frustration, isolation, depression, anxiety, stress, insomnia, anger, and negation (Kang et al., 2020). In particular, in this group of workers, women, workers with more than 10 years of service, and operators who had a history of psychological suffering showed higher risk of stress, anxiety, and depression (Zhu et al., 2020).

A further risk factor for psychological distress has been also a reduced social network support, a protective factor in the stress resilience (Ozbay et al., 2007). In the emergency situation caused by SARS-CoV-2, healthcare workers are indeed at high risk of acute stress, and this risk could be even higher if they feel such a disjunction from the social community formed by the other citizens, as the situation in Italy seemed to lead. In addition to the personal consequences on the psychophysical health of the health professionals, this could easily lead to a progressive decline in their health services, with a worsening of the quality of care provided. The experience with the disease caused by H1N1 in Japan showed how policies that take care of healthcare and give physicians confidence positively affected the overall care they provide to the population (Maunder, 2009; Imai, 2020). For all these reasons, it is very important to study the trait and state psychological variables of healthcare workers as risk or protective factors with respect to the actual stressful situation. In this manner, it would be possible to evaluate the analogies and the differences with the Chinese model at both intracultural and intercultural levels (McCrae, 2001), for considering which intervention strategies could be suited for Italian healthcare workers and thus importing the most adequate recently developed for the Chinese healthcare system in response to the spread of COVID-19.

In such an emergency situation, characterized by contrasts between the opinions and the worries of medical doctors on one side and the behaviors and the attitudes of the general population on the other, we designed and conducted this study. According to the evidences reported above, our objectives were (i) to probe the opinions and the worries relative to COVID-19 spread in both the general population and healthcare workers; (ii) to study which demographic, geographic, and psychological variables were related to a higher perception of the health risks; and lastly (iii) to assess any difference in risk perception relatively to COVID-19 between the general population and healthcare workers. Thus, our aim was to understand the influence of psychological and training/working experience in shaping opinions, worries, and risk perception relatively to COVID-19. To this aim, we administered an online battery including a questionnaire about the direct experience, the opinions and the worries relative to the COVID-19, and some questionnaires evaluating the psychological distress state. To evaluate the participants distress level, we administered questionnaires measuring perceived stress, anxiety, and death anxiety as they usually increased in the general population (Brooks et al., 2020) and in healthcare workers (Brady, 2015; Kang et al., 2020) during emergency situation. As the social-health situation in Italy was evolving continuously in the beginning of March, we limited the data collection in the days 10–12 March 2020.

Based on the evidence reviewed so far, we hypothesized that healthcare workers would show higher levels of distress in terms of stress, anxiety, and death anxiety, particularly in North Italy, where the contagion was higher. In fact, as reported by Lai et al. (2020), direct exposition to virus outbreak affected the psychological health of healthcare workers, with those living in the region of Wuhan reporting higher distress than colleagues living elsewhere. Then, we hypothesized that healthcare workers would perceive higher levels of risk for themselves and for their relatives and that this effect would be true even when controlling for such psychological distress. In fact, we expected that this higher risk perception was not linked only to a worse psychological state, but also to a greater knowledge of the COVID-19 disease and of its possible consequences. Thus, we also expected that healthcare workers would report higher levels of knowledge of the new coronavirus. About containment and prevention measures, we expected that healthcare workers would report a higher engagement in preventing measures with respect to other people and request for more stringent containment measures, in order to prevent SSN collapse due to an increased number of accesses in hospital. Following all the previous hypotheses, we expected that participants not in the healthcare workers group would provide more optimistic forecast about the progress of the spread of COVID-19.

## Materials and Methods

### Participants

Three hundred fifty-three Italian adults participated in this study (mean age = 38.26 years, *SD* = 12.24 years; females = 265, males = 88). We divided our sample by means of their job or training: in the first group, we included medical doctors, nurses, paramedics, and students in medicine/nursing/other medical disciplines (“MED” group; *n* = 167; mean age = 35.56 years, *SD* = 9.90 years; female = 133, males = 34), whereas in the second group, we included all the other participants (no-med or “NOM” group; *n* = 186; mean age = 40.69 years, *SD* = 13.58 years; females = 132, males = 54).

### Procedure

We recruited our participants with a convenience sample method via email and social media. Participants received a brief description of the study together with an informed consent module. After providing the informed consent, they completed an online battery of questionnaires, as described afterward. Data were collected in anonymous format, and participants were invited at the end of the battery to leave their email in order to be contacted for possible follow-up measures. In this study, we collected data not reported here, as fully specified in the “Materials and Methods” section.

### Materials

In this study, we administered questionnaires to evaluate the psychological condition and personality traits of each participant. Where possible, we opted for short or brief version of each questionnaire, in order to contain the total number of items (45 total items). We included in our battery the following questionnaires:

• The four-item Perceived Stress Scale (PSS; Cohen et al., 2006), a questionnaire evaluating the stress perceived by the participant in the last month, that is, the participant’s perceived feeling to be in control over external events, relationships, and emotional life. We used the short four-item version. Each item was evaluated on a five-point Likert scale ranging from 0 (never) to 4 (very often). In our sample, the four-item PSS showed a good reliability score, Cronbach’s α = 0.73, similar to what was reported in the original version, α = 0.72.
• The six-item version of State-Trait Anxiety Inventory (STAI; Marteau and Bekker, 1992), which assessed the anxiety of the participants on six items including emotions or feelings. Each item was evaluated on a four-point Likert scale ranging from 1 (almost never) to 4 (almost always). In our sample, the six-item STAI showed a similar reliability score, Cronbach’s α = 0.85, to that reported in the original version, α = 0.82.
• The death anxiety scale of the Existential Concerns Questionnaire (ECQ; van Bruggen et al., 2017), which evaluated the anxiety of the participant relatively to his/her sense of finitude, to the fear of diseases and death. The total score was computed overall five items. Each item was evaluated on a five-point Likert scale ranging from 0 (never) to 4 (always). In our sample, the ECQ death anxiety scale showed a good reliability score, Cronbach’s α = 0.89 (in the original version, only the internal consistency for the global score was reported, α = 0.92).
• The Marlowe and Crowne social desirability scale (M&C; Manganelli Rattazzi et al., 2000), which assessed the tendency of answering in a socially desirable manner. This version of the scale implied nine items evaluated over a six-point Likert scale ranging from 1 (absolutely false) to 6 (absolutely true). In our sample, the M&C scale showed an acceptable reliability score, Cronbach’s α = 0.62, slightly lower than that reported in the cited Italian validation, α = 0.69.

We also included in the battery other questionnaires, which results were not reported in the present work: the 10-item Big Five Questionnaire (Guido et al., 2015), the Acceptance and Action Questionnaire II (Pennato et al., 2013), and the Emotion Regulation Questionnaire (Balzarotti et al., 2010).

We further developed a questionnaire about SARS-CoV-2 and COVID-19–related experience and personal opinion. Both authors (L.S. and C.G.) compiled a first list of items, and then this list was revised by five experts (medical doctors and psychotherapists) in order to remove, change, or add relevant items. We obtained a final list including 68 items. A complete list of the items was reported in **Appendix A**. To keep the questionnaire simple and easy to understand, we preferred to include mostly yes/no questions. The questionnaire we administered included the following:

• Demographic and personal information, i.e., age, sex, living area in Italy (North, Center, or South), years of study, job, relationship status, number of children, if pregnant or with a pregnant partner, number of cigarettes per day, alcohol drinking, presence (and type) of a chronic disease or other preexistent illness, drugs taken, religious belief, and if vaccinated for flu in 2019;
• Direct experience with the COVID-19 infection, i.e., if tested with the swab, if positive, if COVID-19 symptoms were experienced;
• Preoccupation about infection, at personal, familiar, and social level;
• Opinion about personal and other people’s behaviors since the COVID-19 breakthrough;
• Opinion about the containment measures adopted by the Italian Government; and
• Information received about the disease and the social situation relative to the breakthrough of COVID-19.

### Data Analysis

Data analysis was conducted with statistical software R, version 3.6.3 (R Core Team, 2014). As first step, we assessed differences in our sample between the MED and NOM groups for the demographic variables in order to control for unbalanced factors in our sample. We conducted these comparisons by means of *t*-tests for numerical data and of χ^2^ tests for frequencies. Then, we described the experiences about COVID-19 infection in our sample and compared MED and NOM groups again and areas (North vs. Center vs. South Italy). We also compared the psychological state of our participants by group and area to assess difference in levels of anxiety, stress, and death anxiety. For these comparisons, we implied mixed-effects analyses of variance (ANOVAs) with one between factor (group, two levels: MED vs. NOM) and one within factor (area, three levels: North, Center, South). We further decomposed significant main or interaction effects by means of least significant difference–corrected *post hoc* pairwise comparisons.

As main analysis, we computed hierarchical logistic regression on the dichotomic responses and reported overall our participants about preoccupations, opinions, and behaviors relatively to their experience with the new coronavirus. This analysis allowed us to estimate the odds to obtain a positive response to a particular question given a set of parameters. For non-dichotomous variables (e.g., contagious spread in the next days could either increase, decrease, or stay stable), we created *N* dichotomous dummy variables, where *N* was the number of possible alternative responses to “equal” response (e.g., for contagious spread in the next days, we created a dummy variable for increased forecast and a dummy variable for decreased forecast). We used as reference the middle-point response, i.e., “equal” response, and evaluated the propension to respond “more” or “less” with respect to this point. Moreover, we did not analyze the questions for which we obtained identical or almost identical responses by all our participants, i.e., question with >98% of equal responses. In fact, for such questions, it was easy to find one of the outcome categories so underrepresented that it could lead to rare event outcome or be linearly separated by only one of the independent variables (IVs).

We introduced the regressors in the model at different steps of computation. At the first step, we introduced the demographic variables such as sex, age, and living area (with the North Italy as reference). At the second step, we added to these variables the psychological state factors of perceived stress (PSS score), anxiety (STAI score), and death anxiety (ECQ score), in order to investigate the contribution of these regressors. As last step, we investigated the difference between MED and NOM groups in responding to the questionnaire. For this aim, at the third step, we introduced the group variable as regressor.

When conducting logistic regression analysis, we should check for assumption violations. First, we considered the sample size issue. In the full model, i.e., model at Step 3, we had a total of eight IVs including all the regressors and the covariates. Considering our sample size of 353 participants, this resulted in an event per variable (EPV) of approximately 50, computed as the ratio between number of participants and number of IVs. This EPV could be considered as fairly sufficient to make the interpretation of our global model meaningful (Harrell, 2015; Ogundimu et al., 2016), even if the more stringent Bujang et al.’s rule of thumb Bujang et al. (2018) would suggest to include at least 450 participants for such a number of variables. Moreover, for each tested model, we checked for influential outliers and for multicollinearity. To test for influential outliers, we computed Cook’s distance for each data point and check for values larger than 3 SD from the mean, as a large value of Cook’s distance indicates an influential observation (Martín and Pardo, 2009; Zhang, 2016). To test multicollinearity, we computed the variance inflation factor (VIF) for each regressor and check for any value greater than 2.5, considered as more strict threshold with respect to the usual value of 5 or 10 (Midi et al., 2010). For all our logistic regression models, we found no influential outliers or any VIFs greater than the threshold value. The results of these tests, together with the reported EPV greater than 50, testified that our logistic regression analyses could be considered sufficiently reliable.

To further support our logistic regression model results, we conducted semipartial correlation analysis by means of the *ppcor* package for R (Kim, 2015). We assessed the degree of relationship between group (coded as NOM = 0 and MED = 1) and each dependent variable of the COVID-19 questionnaire while controlling for sex, age, living area, anxiety, death anxiety, and stress. Semipartial correlations were reported as Pearson *r* for each computed correlation, with values ranging from −1, very strong negative relationship, to 1, very strong positive relationship.

Even if we conducted a great number of statistical analyses on the same sample, we decided not to apply a general correction to significance level for multiple tests. Because of the exploratory nature of this study, we preferred not to strictly control over false-positive rate (Type I error) while avoiding to inflate false-negative rate (Type II error); i.e., we decided to collect all the significant results emerging from our analysis so to guide further, confirmatory experiments and studies (see Fiedler et al., 2012, for an overview of the problem on multiple testing correction).

## Results

Table 1 reports the descriptive statistics for the two groups and the relative tests for samples’ comparison. As shown, participants in the MED group were younger (mean = 35.56 vs. 40.69), studied more years (mean = 23.02 vs. 21.34), had less children (mean = 0.40 vs. 0.58), reported to sleep in average less time per night (mean = 6.84 vs. 7.06), and were more frequently vaccinated for annual flu in 2019 (40% vs. 13%).

**TABLE 1:** Descriptive statistics computed overall the sample and for the two groups separately.

Variable	Overall (*n* = 353)		MED group (*n* = 167)		NOM group (*n* = 186)		Statistical comparison
	Mean	*SD*	Mean	*SD*	Mean	*SD*	
Age	38.26	12.24	35.56	9.91	40.69	13.58	*t* (351) = 4.02*
Years of study	22.14	5.07	23.02	4.66	21.34	5.30	*t* (351) = −3.15*
Children	0.49	0.80	0.40	0.73	0.58	0.85	*t* (351) = −2.19*
Sleep hours per night	6.96	0.92	6.84	0.94	7.06	0.89	*t* (351) = 2.24*
Number of cigarettes per day	2.20	4.77	2.11	4.49	2.27	5.03	*t* (351) = 0.32
Alcohol consumption (1–4)	0.95	0.73	0.90	0.73	1.00	0.73	*t* (351) = 1.31

	**Proportion**		**Proportion**		**Proportion**		

Sex	0.75		0.80		0.71		χ^2^ (1) = 0.01
In a relationship	0.68		0.68		0.69		χ^2^ (1) = 0.93
Pregnant (or pregnant partner)	0.04		0.04		0.03		χ^2^ (1) = 0.08
Religion (catholic or others)	0.46		0.46		0.47		χ^2^ (1) = 0.61
Chronic disease/illness	0.27		0.28		0.27		χ^2^ (1) = 0.17
Flu vaccine in 2019	0.25		0.40		0.13		χ^2^ (1) = 19.60*
Italy area							
North	0.18		0.14		0.23		χ^2^ (1) = 6.50*
Center	0.63		0.70		0.55		χ^2^ (1) = 2.25
South	0.19		0.16		0.22		χ^2^ (1) = 0.73

Comparisons were conducted by means of t test for numerical variables and of χ^2^ test for categorical variables. *p < 0.05.

### Experience With the COVID-19

In this first results section, we reported the analysis of the data relatively to the experience with the COVID-19. We thus referred to the data in the first part of the questionnaire, in which we asked if participants had personal experiences or contacts with COVID-19 infection. We reported data overall participants and divided by groups in Table 2. Frequencies were compared by means of χ^2^ test.

**TABLE 2:** Frequency (in%) of “*y*es” responses to each question, computed by area and by group.

No.	Question	Area (overall sample)			Group	
		Center	North	South	NOM	MED
(1)	Have you done a throat swab for SARS-CoV-2?	0.00	3.13	0.00	0.00	1.19
(2)	If yes, was it positive?	0.00	1.56	0.00	0.00	0.59
(3)	Do you or have you recently had one or more symptoms related to COVID-19?	36.94	42.19	40.30	36.02	41.32
(4)	If yes, did you think could be COVID-19?	6.76	18.75	1.49	5.91	10.18
(5)	If yes, have you alerted the national health service?	2.70	7.81	0.00	2.63	3.59
(6)	Are you currently or have you been on spontaneous or imposed quarantine for COVID-19?	22.97	31.25	17.91	26.34	20.36
(7)	Are you currently or have you recently been in contact with people at high infectious risk?	22.07	53.13	17.91	15.59	39.52
(8)	Are you currently or have you recently been in contact with people who had a positive test for COVID-19?	4.05	14.06	2.99	0.00	11.98
(9)	Have any positive cases of COVID-19 infection been detected in your living area or city?	88.29	98.44	68.66	83.33	89.82

For the overall sample, we found an effect of the living area on question 3, about the presence of symptoms related to COVID-19, χ^2^(2) = 44.48, *p* < 0.01; question 4, about thinking that the symptoms relate to a COVID-19 infection, χ^2^(2) = 11.64, *p* < 0.01; question 6, about the quarantine status, χ^2^(2) = 30.67, *p* < 0.01; question 7, about contact with people at risk of infection, χ^2^(2) = 21.87, *p* < 0.01; and question 9, about the presence of positive case in the living area or city, χ^2^(2) = 132.71, *p* < 0.01. In answering to all these questions, participants from North Italy reported a greater direct experience with COVID-19 than participants from Center or South Italy, whereas participants from Center Italy reported more personal experiences than participants from the South.

Then, we compared the frequencies between the two groups, MED versus NOM. We found significant differences in question 7, about contact with people at risk of infection, χ^2^(1) = 14.41, *p* < 0.01, and in question 8, about contact with people positive for COVID-19 test, χ^2^(1) = 20.01, *p* < 0.01, with participants in the MED group reporting more frequent contacts with people at high risk of infection or already positive.

### Comparing Psychological Variables Between Groups

We measured various indexes of psychological distress state, i.e., anxiety, death anxiety, and stress. Here, we tested if any difference existed between groups in the psychological state and if this difference was modulated by the living area. To this aim, we conducted mixed-effects (ANOVAs) with a between-variable of group (MED vs. NOM) and a within-variable of living area (North vs. Center vs. South Italy). We controlled for the effect of age and sex as covariates. We probed significant effects by means of *post hoc* corrected tests.

**FIGURE 1:**
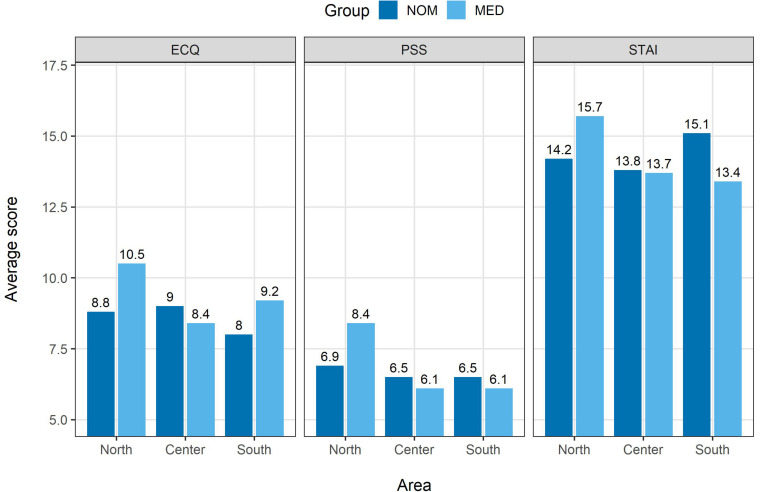
Average score for ECQ **(left)**, PSS (middle panel), and STAI **(right)** plotted by group (MED vs. NOM) and area (North, Center, or South). Average scores for each combination of group and area are reported on top of the bars.

For the death anxiety score (ECQ; see Figure 1, left panel), we found no significant main effects or significant interaction, all *p*’s > 0.19. For the Perceived Stress Score (PSS; see Figure 1, middle panel), we found a significant main effect of the living area, *F*(2,348) = 6.52, *p* < 0.01, with participants from North Italy reporting higher stress levels than participants from both Center, *p* < 0.01, and South Italy, *p* < 0.01. The analysis also revealed a significant group × living area interaction, *F*(5,345) = 3.16, *p* < 0.05, with MED participants from North reporting higher stress score than other MED participants from both Center, *p* < 0.01, and South Italy, *p* < 0.01, as well as higher stress score than the NOM group participants from all living areas, all *p*’s < 0.05. For the anxiety score (STAI; Figure 1, right panel), we found a significant main effect of living area, *F*(2,348) = 3.31, *p* < 0.05, with participants from North Italy reporting higher anxiety levels than participants from Center, and a significant group × living area interaction, *F*(5,345) = 2.96, *p* < 0.05. The interaction was due to a significant difference in anxiety between MED participants from North with respect to the MED participants from Center and South Italy, *p*’s < 0.01, and with respect to NOM participants from Center Italy, *p* < 0.01. This analysis thus revealed that the MED group participants from North Italy reported higher levels of anxiety and stress than the general population and the medical and paramedical staff from other living areas.

### Descriptive Statistics of the Questionnaire About COVID-19

Before conducting the regression analysis on the questionnaire data, we reported some descriptive information and statistics about the response frequency of participants. Response frequencies for each item overall sample as well as divided by group are reported in Table 3, left group of columns. Here we reported also a χ^2^ test comparing the frequency of “yes” responses for the two groups.

**TABLE 3:** Hierarchical logistic regression odds for demographics (Step 1), psychological (Step 2), and group (Step 3) factors for the COVID-19 questionnaire.

Question	Frequency of “yes” responses				Step 1				Step 2			Step 3	
	All	MED	NOM	χ ^2^ test	Sex	Age	Area Center	Area South	PSS	STAI	ECQ	MED	Sp. cor.
Do you think you are currently at infectious risk?	0.57	0.71	0.45	5.72*	1.68*	0.99	0.46**	0.64	1.11*	0.95	1.02	2.71**	0.23**
Did you think you were at risk when the first cases appeared in Italy in January 2020?	0.18	0.23	0.14	2.25	1.02	1.02	0.38**	0.41*	1.08	0.99	1.03	1.94*	0.13*
Do you think your family members/loved ones are currently at infectious risk?	0.65	0.73	0.57	1.12	1.71*	0.98**	0.59^+^	1.21	1.05	1.03	1.00	1.70*	0.13*
Are you worried about the possibility that, in case of infection, you may have serious complications or die?	0.57	0.59	0.55	0.08	2.99**	1.03**	1.57	1.30	1.03	0.98	1.16**	1.33	0.04
Are you worried about the possibility that, in case of infection, some of your family/loved ones may have even serious complications or die?	0.93	0.95	0.92	0.51	2.10^+^	0.98	0.01	0.01	0.95	1.02	1.11*	0.98	0.02
Are you worried about the possibility that the situation may precipitate at global level in the near future due to COVID-19?	0.73	0.76	0.70	0.03	1.88*	1.01	0.72	1.09	1.06	1.03	1.05^+^	1.33	0.06
Are you worried about the possibility that, if the national health system was unable to guarantee treatment or to support the volume of hospitalized patients, episodes of violence and abuse may occur among patients or their families?	0.83	0.86	0.81	0.17	1.49	0.97**	0.94	1.06	1.10	1.11^+^	1.02	1.27	0.04
Are you concerned about the possibility that other people’s behavior in response to this situation could be more dangerous than the medical risks associated with COVID-19 infection?	0.83	0.86	0.81	0.12	1.89*	0.95**	0.87	0.98	1.06	1.01	1.00	1.08	0.02
If you work in the medical/health sector, do you fear that the scarcity of means and resources of care foreseen for the near future could expose you to episodes of violence or retaliation by patients or their families? (answer “no” if you are not a doctor/other health worker)	0.73	0.73	–	–	0.98	1.00	0.53	1.11	1.00	1.05	1.06	–	–
Do you think you might have put yourself at risk of infecting yourself with your behavior?	0.18	0.23	0.15	1.86	1.68	0.97*	0.60	0.51	1.08	0.94	1.05	1.34	0.06
Do you think you might have put yourself at risk of infecting your family/loved ones with your behavior?	0.17	0.22	0.13	2.40	2.23^+^	0.96**	0.55	1.02	1.04	1.00	1.03	2.68	0.06
*Are you implementing the hygienic–sanitary prevention provisions such as washing your hands often, avoiding physical contacts (handshakes, kisses, and hugs), sanitizing surfaces, keeping a certain distance from the interlocutors?*	0.97	0.98	0.97	0.74	–	–	–	–	–	–	–	–	–
Do you think people are having appropriate behaviors for the situation?	0.11	0.07	0.16	8.10**	1.05	1.03*	2.03	2.19	1.09	0.93	0.96	1.13	-0.12*
Are you worried/angry about the violation of containment provisions shown by some people in the last few days?	0.89	0.90	0.88	0.62	2.65**	1.02	0.51	0.46	1.04	0.89^+^	1.07^+^	1.43	0.02
Do you think it would be right to punish more severely public health risky behaviors?	0.90	0.92	0.88	0.32	3.37**	1.00	1.19	1.86	1.04	1.02	1.04	0.46	0.05
Are you continuing or have you continued in the last few days to attend meeting places for recreational purposes such as pubs, restaurants, malls, fairs, events, cinemas, or theaters?	0.06	0.04	0.07	2.33	0.32**	0.95*	1.52	0.36	0.99	1.04	0.96	0.86	-0.08
Have you recently bought more canned, long-life food and/or bottled water to stock up on it?	0.23	0.22	0.24	0.80	1.75^+^	1.01	0.98	1.06	0.96	1.05	1.00	2.56	-0.03
*If the virus spread in your living area, would you try in any way to move to an area considered safer?*	0.02	0.02	0.02	0.01	–	–	–	–	–	–	–	–	–
Do you think concern and fear surrounding COVID-19 are valid reasons to violate the sanitary containment provisions?	0.71	0.74	0.67	0.01	0.89	1.00	1.08	0.47*	0.98	1.00	1.00	1.65*	-0.05
Do you think the national health system would currently be able to take care of you if you got infected?	0.71	0.84	0.60	3.57	1.12	1.00	0.80	0.97	1.03	0.96	1.01	3.78**	0.09
Do you think it is right to give care priority to people with greater hope of survival in case of need or shortage of hospital beds?	0.25	0.26	0.23	0.01	1.12	1.01	0.64	0.94	1.06	0.92^+^	0.98	1.19	0.27**
If you or one of your family/loved ones were prevented from accessing to intensive care to give priority to patients with a higher probability of survival, would you accept this decision at all kindly?	0.05	0.04	0.05	1.00	0.72	0.99	1.69	1.65	0.93	1.17^+^	1.08	0.65	0.05
*Do you think that virus containment measures are necessary?*	0.98	0.99	0.97	0.65	–	–	–	–	–	–	–	–	–
Do you judge the current containment action as adequate?	0.56	0.51	0.61	3.96*	0.85	1.01	2.41**	2.74**	0.98	0.98	0.98	0.72	-0.09
Do you think that the containment measures need to be improved or strengthened?	0.80	0.84	0.76	0.01	1.90*	1.00	0.74	0.81	0.98	1.01	1.04	1.63^+^	0.09^+^
Do you think it is right to use the army or the public force to enforce health containment measures?	0.91	0.93	0.89	0.31	0.97	1.02	1.08	0.91	1.06	1.04	1.01	1.90	0.08
Do you think it is right to limit people’s freedom in view of greater virus containment?	0.93	0.96	0.91	0.30	4.71**	1.01	1.80	1.28	1.00	1.00	1.05	2.25	0.07
*Do you think it is right to limit your risky behaviors autonomously (for example, avoid leisure travel, do not attend crowded places, do not participate in events)?*	0.99	0.99	0.99	0.93	–	–	–	–	–	–	–	–	–
*Are you currently limiting your risky behavior?*	0.99	1.00	0.97	0.56	–	–	–	–	–	–	–	–	–
Do you think you are properly informed about the characteristics of COVID-19?	0.84	0.93	0.76	0.57	1.39	1.01	0.89	1.29	0.90	1.02	0.99	4.53**	0.24**
Do you think you are properly informed about the political/social situation related to COVID-19?	0.7	0.77	0.65	0.26	0.83	1.00	0.57^+^	0.50^+^	0.92	1.02	1.04	1.97**	0.15*
Do you think more communication from experts (such as virologists and other doctors) is needed?	0.68	0.69	0.68	0.50	0.75	0.96**	1.53	1.68	1.14**	0.94	0.97	0.92	-0.03
Do you think that media are too much or too insistently concerned with COVID-19?	0.57	0.59	0.55	0.08	1.40	0.99	0.85	1.14	1.04	1.02	0.99	1.07	0.02
Do you think there is any sensitive information, related to COVID-19, hidden from you?	0.40	0.34	0.46	5.52*	1.13	1.00	1.69^+^	2.23*	1.04	1.00	0.99	0.60*	-0.12*
If yes, do you think they are related to a real greater danger of the virus? (if you answered “no” to the previous question, select “no”)	0.68	0.77	0.62	0.84	1.47	1.04*	1.28	1.65	0.98	1.11	0.96	2.70*	0.18*
	**According response (to “equal” reference)**												
Spread of the virus will slow down in the next few days	0.35	0.19	0.45	15.36**	1.27	1.03^+^	0.92	1.32	1.01	1.00	0.99	0.28**	-0.23*
Spread of the virus will accelerate in the next few days	0.73	0.75	0.72	0.53	1.37	1.01	0.75	0.98	0.98	1.04	0.97	1.20	0.05
Spread of the virus will slow down in the next few weeks	0.75	0.71	0.78	5.49*	0.97	1.00	1.23	1.52	0.87*	1.07	1.02	0.71	-0.07
Spread of the virus will accelerate in the next few weeks	0.71	0.73	0.69	1.07	1.52	1.00	1.27	1.94	0.85*	1.14*	1.02	1.31	0.05
Perception of risk related to COVID-19 in public opinion is lesser than it should be	0.64	0.72	0.56	1.67	1.80*	0.99	0.50^+^	0.56	0.99	0.97	1.02	1.83*	0.14*
Perception of risk related to COVID-19 in public opinion is greater than it should be	0.30	0.33	0.29	1.33	0.70	0.95**	0.41^+^	0.75	0.88	1.00	1.04	0.96	-0.01

Questions in italics showed imbalanced responses (almost all “yes” or “no”). Sex was coded as 0 = male, 1 = female. Group was coded as 0 = NOM and 1 = MED. ECQ = Existential Concerns Questionnaire (death anxiety scale); PSS = Perceived Stress Scale; STAI = State-Trait Anxiety Inventory. Rightmost column (Sp. cor.) reports Pearson r for semipartial correlations between group and questionnaire responses (coded as 0 = “no” and 1 = “yes”) controlling for all the other variables, i.e., sex, age, living area, anxiety, death anxiety, and stress. Significance level marked as follows: ^+^p < 0.10, *p < 0.05, **p < 0.01.

Of note, 57% of participants were thought to be at risk of contagion, but only 18% were thought to be at risk when the first cases appeared in Italy. They also thought that their loved ones would be at risk (65%). The MED group reported higher frequency of thinking to be at risk (71%). Many participants in this group (57%) were scared about health consequences or death if infected, but almost all (93%) were more worried for family or loved ones’ consequences of infection. Similarly, the 73% of them reported worries about the global sociopolitical implication of virus spread, and the 83% about the possible collapsing of the national health system. Moreover, 83% of them thought that people’s behavior could be ever scaring of the infection and 73% were worried by the increased aggression risk for health workers in the near future.

Most of our sample (97%) reported to adhere to hygiene measures and to avoid public events or places (94%), and only a few participants reported to have risky behaviors for themselves (18%) or their family (17%). However, only 16% of NOM and 6% of MED believed that people’s behavior was adequate to the situation. About violation of the public health dispositions, most of participants thought that violation should be punished more severely (90%) or that the national army should be implied (91%), as they reported to be preoccupied or angry toward such violations (89%). Few reported to have bought more canned food (23%), and very few participants reported that they would try to escape if the infection would spread in their living area (2%), even if a great part of them (71%) believed that the infection fear could be considered a valid reason to break the containment rules.

About the possible problem of accessing healthcare services, most of participants (71%) believed in the national health system, whereas few thought that it was right to give priority to people with greater hope of survival in case of shortage of hospital beds (25%) and even less (5%) that they would accept an exclusion for them or their loved ones at all kindly.

About their opinions on the containment measures disposed, 98% of the participants thought that these measures were necessary, but only 56% thought that these same measures were adequate (the MED group was more skeptical than the NOM group), and 80% proposed to strengthen them. In line with this, most participants thought that it was right to limit people’s freedom for controlling the virus (93%), as well as one’s own freedom (99%), as they already limited their behaviors (99%).

About the information, they reported to be properly informed about the virus (84%) and the social situation related to it (70%), but also requested more information from experts (68%). About perception of risk in public opinion, 64% of participants reported to think that it was lesser than it should be and 30% that it was greater. Interestingly, 40% reported to think that there was some hidden information about the virus, and 68% of these that such hidden information was related to a greater danger related to the infection. The MED group, instead, reported to be less convinced of the existence of hidden information (34% vs. 46% of the NOM group).

Lastly, about the spread of the virus, the MED group was more pessimistic than the NOM group. In fact, they reported less likely that the spread would slow down in some days (19% vs. 45%) or in some weeks (71% vs. 78%).

### Logistic Regression Overall Sample: Effect of Demographics

In this subsection, we present logistic regression results on the COVID-19 questionnaire. We used each question as a dependent variable in a three-step hierarchical logistic regression. At Step 1, we used as regressors the demographic variables (see “Data Analysis” section) and the living area, considering North Italy as the reference (the coefficients reported should be interpreted as the odds that a participant from Center or South Italy would answer “yes” to a question compared to a participant from North Italy). At Step 2, we added as regressors the psychological factors of perceived stress, anxiety, and death anxiety. Finally, at Step 3, we included the group effect. Along with Step 3 results, we also provided semipartial correlation score for the relationship between each dependent variable and the group (coded as 0 = NOM and 1 = MED). For the sake of brevity, we reported only the questions for which we obtained significant regressors.

At Step 1 (see Table 3, Step 1 block of columns), we included in the model only demographic variables. Of these, the most influential were sex and age. With respect to male sex, female sex was linked to higher odds to be concerned by the following risks: being infected (1.68), loved ones being infected (1.71), developing serious complication or dying (2.99), global crisis (1.88), people’s behavior in response of virus outbreak (1.89), infecting family members or love ones (2.38), and people’s violating the containment provisions (2.65). In fact, females had higher odds to report that the public opinion had less risk-related perception about COVID-19 than it should be (1.80), that risky behavior should be punished more severely (3.37), that containment provisions should be improved (1.90), and that it would be right to limit people’s freedom in this situation (4.71). In line with these results, they reported more likely to have not continued to attend public places and events (0.32).

About age factor, older age was related to lower odds of reporting worries about the risk of infection for the loved ones (0.98), or about people’s behavior as more dangerous that virus infection (0.95), or about the perception of risk in public opinion as lower that is should be (0.95). Older age people also reported lower odds to be concerned about their behavior as risky for themselves (0.97) or loved ones (0.96), but higher odds to be concerned about their health status in case of COVID-19 infection (1.03) and by people reaction to virus spreading (1.03). Lastly, they reported lower odds to request for more information by experts on media (0.96).

Also, the living area had a relative impact on the outcome variables at this step. With respect to participants from North Italy, those from both Center and South Italy showed greater odds to judge the actual containment measures as adequate (Center = 2.41, South = 2.74) and to think that some information about COVID-19 was hidden from them (Center = 1.69, South = 2.23), whereas they reported less likely to be at infectious risk (Center = 0.46, South = 0.64 not significant) or to consider themselves at risk when the first cases were discovered in Italy (Center = 0.38, South = 0.41). Of note, participants in South area reported lower odds with respect to participants in North area to consider fear of infection as a valid reason to violate the containment measures (0.47).

### Logistic Regression Overall Sample: Effect of Psychological Factors

At Step 2 (see Table 3, Step 2 block of columns) of hierarchical model, we added psychological factors of perceived stress (PSS), anxiety (STAI), and death anxiety (ECQ). We found that these factors were related to few, but interesting outcomes. In particular, the PSS score was related to a higher worry to be currently at infection risk (1.11) and a major need of information by experts (1.14), while their opinion on the virus spread was that it would show equal speed in the weeks following the compilation (0.84 for both accelerated or slowed-down spread). Instead, the STAI score was related to higher concerns of accelerated spread of virus in the weeks following the compilation of the questionnaire (1.14). Lastly, the ECQ score was related to a higher level of worrying about the COVID-19 situation, in particular about possible severe outcome of the disease for themselves (1.16) or loved ones (1.11) and marginally related to higher level of worrying about possible catastrophic social global outcomes (1.05) or violation of containment measures (1.07).

### Effect of Group on Logistic Regression Model

At Step 3, we added to the logistic regression model the group factor to check for the predictive effect of being in the MED or NOM group while controlling for both demographic and psychological variables. Results are reported in Table 3, Step 3 column (see the rightmost column). Participants in the MED group reported higher odds of thinking to be at actual risk of infection (2.71) and also to be at risk from the beginning of the COVID-19 spread in Italy (1.94). They also reported more likely to think that their family or loved ones were at risk of infection (1.70). The MED group showed higher odds to report that the fear of contagion would be a valid reason to violate the containment measures (1.65) and that the SSN would adequately cure them in case of infection (3.78) and to report a sufficient level of information about the characteristics of the disease (4.53) and about the social situation relative to COVID-19 (1.97). They also reported less likely that some information about the virus was hidden (0.60), but the ones who answered affirmatively to this question had more than two times the odds with respect to the NOM group thinking that such hidden information was related to a greater virus-related danger (2.70). About the spreading of the virus, participants in the MED group were less probably convinced that the virus spread would slow down in the following days after the compilation of the questionnaire (0.28). Lastly, the MED group participants more likely reported that perception of risk in public opinion was lower than it should be (1.83).

Semipartial correlations mostly confirmed this pattern of results. However, differently from the logistic regression, this analysis revealed that the MED group was related to the opinion that people’s behavior was not adequate to the situation, *r* = −0.12, and to agree to give care priority to people with greater hope of survival, *r* = 0.27. Also, semipartial correlations did not confirm the regression results for the questions about the fear of contagion as a valid reason to violate the containment measures, *r* = −0.05, and the adequacy of the SSN to take care of people in case of infection, *r* = 0.09.

## Discussion

In this article, we investigated the worries and the perception of risk toward the health and social situation in Italy related to the outbreak of COVID-19. To this aim, we conducted a cross-sectional study by means of online questionnaires administered to a convenience sample of volunteer participants including both health workers and the general population. We asked participants to report their worries and opinions about COVID-19 in about 50 different questions combined with psychological variables measuring stress, anxiety, and death anxiety. We obtained and analyzed data from 353 Italian adult, divided in 167 participants in the MED group (medical doctors, paramedics, health workers, and students) and 186 participants in the NOM group. We mainly compared the answers given to the questionnaires by these two groups. We also investigated the effect of the living area in Italy, as the northern regions were more involved than the central and southern ones (Cereda et al., 2020).

### Anxiety and Stress as Related to Living Area and Job

First, we assessed risky situations in which people were involved relatively to COVID-19. As expected, people from North Italy reported more direct experiences with COVID-19, including more symptoms related to the infection, more prolonged quarantine status, more contacts with people at risk, and higher numbers of positive cases in their zone. The MED group, instead, reported a higher number of contacts with people currently infected or at risk. Thus, both living area and group predicted a major or minor probability to be involved in risky situations or contacts. Following this, we found that participants from North Italy reported higher levels of stress and anxiety and in particular that health workers in North area showed a higher level of both health workers from other areas and the general population from the same area. Thus, both living area and job combined with the higher exposition to infection risk in order to increase the level of stress and anxiety in health workers from North Italy.

We would caution about the relatively small number of participants in each area divided by group: our results about living area should be considered strictly as preliminary. Further studies are welcome in order to confirm or refute the results that we presented on this topic. However, we should note that our result was in line with the psychological response of health workers in China, where Lai et al. (2020) found that psychological distress increased for workers closer to the outbreak of epidemic (i.e., who lived and worked in Wuhan region) or assigned to patients affected by COVID-19. Thus, the same rule applies here: the closer to the risk of infection, the higher the risk of acute psychological distress.

Similar results were found in previous researches on new disease outbreaks. For example, Wong et al. (2007) reported higher levels of anxiety in university students during the SARS epidemic, in particular among medicine students and students living in the area in which the infection spread more. Also Wheaton et al. (2012) reported higher levels of anxiety in students in response to pandemic spread of H1N1. More generally, anxiety emerged in response to various viral diseases, from the annual influenza virus to the H1N1 pandemic (Coughlin, 2012). In the period of viruses spread, anxiety seems to increase in population along with mood disorders, and this increase was related to exposition and infection risk. In line with these results, participants of our study reported higher levels of perceived stress and of anxiety proportional to their risk of infection, i.e., health workers from North were more stressed and anxious than both their colleagues in Center and South Italy and the general population.

While our result supports an acute increase of stress and anxiety, we should carefully monitor the psychological state evolution in order to assess also the effect of COVID-19 over time. In fact, we expected that the virus spread and the quarantine state endurance in Italy could have also mid- and long-term consequences. Survivors from SARS reported posttraumatic stress, anxiety, and depression symptoms 1 month after discharge, suggesting that life-threating condition could have important psychic sequelae (Wu et al., 2005). Such sequelae could be even more significant in health workers, showing higher levels of psychological distress both during and after a quarantine period (Brooks et al., 2020). For this reason, supporting psychological intervention for healthcare workers could be crucial in the first phase of an outbreak (Xiang et al., 2020), in particular considering that a timely and effective intervention could greatly reduce the later onset of posttraumatic stress disorder symptoms following a catastrophic event (Watson et al., 2002).

### Risk Perception and Worries About COVID-19

We analyzed the answers to our questionnaire on COVID-19 by means of logistic regression. For each item, we computed the response odds related to each regressor in three successive steps, by adding sequentially demographic factors, psychological factors, and the group factor. Here we discussed the implication of all these computational steps by dividing the questionnaire items by content. In this section, we discuss the variable that we found for the items relatively to risk perception and worries related to the COVID-19 outbreak in Italy.

Group was strongly related to risk perception: healthcare workers showed about 2.5 times the odds of other participants to perceive themselves at risk of infection, as well as about two times the odds to think they were at risk even at the very start of virus outbreak in Italy. Moreover, they worried about their family situation and about virus spread as they reported that it would not slow down. This supported the idea that medical doctors, nurses, and paramedics had greater risk perception about the COVID-19 infection, probably due to also a greater exposition to danger and to suspect positive cases. Also, living area predicted the perception of risk, as both participants from Center and South Italy reported 0.5 times less preoccupation about risk of infection with respect to participants from North Italy. Again, combination of work, i.e., health workers, and area, i.e., North Italy, combined for the greater perception risk.

About the demographics, the stronger regressor of such worries was female sex, which was related to higher perception of risk, both at personal and family levels, and of a number of worries about social situation and people’s behaviors. In particular, female healthcare workers were reported to be at higher risks of stress, anxiety, and depression during the COVID-19 outbreak in China (Lai et al., 2020; Zhu et al., 2020). This increased distress level in female health workers could be related to an increased perception of risk for themselves and for their relatives as we found in our study, as also reported usually in researches about risk perception in female participants (Gustafsod, 1998). Our results suggested carefully supporting female healthcare workers implied in COVID-19 treatments, as they could be more exposed to risk-related stress compared to their male colleagues. Another important demographic variable was age, as we found that aged people were more worried than younger people about severe consequences of COVID-19, as they already knew that the disease was more dangerous for older people, in particular when older than 60 years (Novel Coronavirus Pneumonia Emergency Response Epidemiology Team, 2020).

Lastly, also psychological factors influenced the odds of perceived risk of infection. In fact, stress was related to increase in perceived risk, while death anxiety was related to the concern about fatal or severe consequences of COVID-19. While the effect of both stress (Traczyk et al., 2015; Sobkow et al., 2016) and death anxiety (Langford, 2002) on risk perception and risk taking was already reported in literature, it should be noted that higher levels of stress could also be due to actual exposure to contagion risk in the case of COVID-19, as shown by our results about comparisons on levels of perceived stress between healthcare workers from North, Center, and South Italy.

Taken together, all these results suggested a higher risk perception relative to COVID-19 in healthcare workers living in outbreak areas, especially if females and with high levels of stress. For COVID-19, knowledge on medicine and on virus could thus increase risk perception, whereas in other fields such as nuclear radiation usually knowledge was associated to lower risk perception (e.g., Sjöberg and Drottz-Sjöberg, 1991). It should be noted that, in case of nuclear radiation, knowledge could be associated to an increase capacity of avoiding risky behavior or situations, whereas in case of COVID-19 spread knowing, the health risks related to disease, but feeling powerless against its containment could exacerbate the danger perception. A reducing stress intervention by means of psychological support to medical workers could reduce the worries due to the perceived risk, so that they could avoid both risky behaviors and overwhelming, stressful concerns.

### COVID-19–Related Behaviors and Containment Actions

We proceed here by discussing the variables related to risky behaviors, judgments about behaviors, and confinement actions. In this respect, female participants reported higher levels of worries about their own behavior, as well as other people’s behaviors as risky. Related to this, they also were four times more likely than men to report the thought that it would be right to limit people’s freedom in order to block the virus spread and three times more likely than men to request more severe punishment for risky behaviors. Capraro and Sippel (2017) showed that females adopted stricter moral judgments than men in personal dilemmas, such as behaving appropriately in the actual COVID-19 outbreak scenario. Females seemed more prone to strict adherence to rules and even to imply stricter rules, probably also in relationship to their increased perception of risk (see section “Risk Perception and Worries About COVID-19”).

Also, the living area showed a strong relationship with these dependent variables. Participants from Center and South Italy were more likely to judge the containment measures as adequate compared to participants from North Italy. On note, participants from South also reported less likely than North ones that concerns about COVID-19 were a valid reason to violate the containment measures. This result could be related to the recent great “escape” of people from the North Italy (when virus spread initially) toward the South, increasing worries in South population, politicians, and medical staff. Again, please consider results on living area no more than preliminary because of the limited number of participants per area in our sample.

Lastly, we should mention that both the group variable and the psychological factors had none or little impact on these variables Thus, our data suggest that opinions and judgments about behaviors and containment actions rely more on demographic variables than on psychological or work-related ones.

### Perceived Knowledge of COVID-19–Related Information

In this section, we discuss how demographic, psychological, and group variables impacted on the perceived level of knowledge relative to COVID-19 and to its related sociopolitical situation. In this regard, the group was the strongest factor. In fact, healthcare workers reported higher odds than non-medical participants of being properly informed about both COVID-19 and its related social situation. They also were less likely convinced that some information about coronavirus was hidden from public opinion, but those who credited such secret information more likely believed that this information was about a greater virus threat. Also, they reported the opinion that perceived risk in the population was not adequate. This result pattern suggests a large gap between the two groups about the perception of being properly informed.

This information gap could explain the risk perception difference, because a greater knowledge could actually influence the personal risk awareness. It should be noted that, in general public opinion, the risk related to the new coronavirus was mistakenly considered as similar to that related to the common cold or annual influenza viruses, an error that could have been induced by the similarity in the spreading strategy and of some of the symptoms. This underrepresentation of fatal or serious outcomes of COVID-19 led to a poor adherence to health recommendations in the very first phase of the coronavirus outbreak in Italy, with important consequences afterward. These considerations seem to suggest that the reduction of such an information gap could eventually mitigate the disproportion in risk perception between groups and consequently increase the adherence to public health rules. Also, our results seem to support this possibility because of the lack of information from experts lamented by more stressed participants, who also perceived a higher level of personal risk. To this aim, an information campaign about the novel coronavirus characteristics, its related disease symptoms and consequences, and public health problems linked to that could greatly support population in this moment, reducing the stress and also the risky behaviors.

However, increasing the communication and the information could not be the most appropriated solution to the problem. In the last decades, especially because almost everyone has a large access to internet resources, we have witnessed not only a significant spreading of online information, but also misinformation; this is causing the diffusion of baseless rumors, difficult to erase from common people system of beliefs (Kata, 2010; Del Vicario et al., 2016). Misinformation spreading combines with people’s distrust in experts’ authority, a more and more rising phenomenon despite the increase in the general education level. As a result, as proposed by Gawande (Gawande, 2016, p. 3): “to defend those beliefs, few dismiss the authority of science. They dismiss the authority of the scientific community. People do not argue back by claiming divine authority anymore. They argue back by claiming to have the truer scientific authority.” This kind of problem is well known in the field of the unfounded, yet persisting, vaccine fear. In anti-vaccination movement, this mistrust phenomenon has been also exasperated by conspiracy theorists and other actors moving criticisms toward physicians and other experts, accused of having conflict of interests or searching media visibility. The same criticisms, however, are often not applied, for the antiscientific community, to the studies supporting their theories (Kata, 2010). All these factors could have an effect also on the underestimation of medical advices and warning on COVID-19 infection by the general population, resulting in the unappropriated behaviors expressed. Thus, providing more information to population could be ineffective, if not supported by psychological evaluation of social dynamics underlying the antiscientific phenomenon, for example, the questioning of the legitimacy of traditional authorities (see Kata, 2010). Understanding how to contrast such a phenomenon could be even more important in case a vaccine for COVID-19 is provided, as already happened for the H1N1 flu in 2009, when many people refused to vaccinate despite the availability of a vaccine (see Offit, 2009). Further studies are needed in order to investigate these contrasting hypotheses for planning effective interventions relative to public health problems.

### Limitations and Future Directions

This study is not free from limitations. First, it implied a cross-sectional design; thus, a relationship between variables could be interpreted only with cautions. Second, we implied a convenience sample method to recruit our volunteer participants, with a possibility for introducing biases that could undermine the possibility to generalize our results to the entire population. We also collected a small sample with respect to the optimal one, i.e., about 450 participants (as suggested by Bujang et al., 2018), thus calling for caution while interpreting our results. For all these reasons, we should underline that our results could not be considered as conclusive and they should be confirmed with further experiments or studies. However, we should note that we conducted this study with two major difficulties. The first was a time-related issue: we had a very short time to collect data as the containment rules and the virus spread vary at a day-by-day rate. Thus, we should collect our data in a concise and brief timeframe. The second issue was a logistic one: most people in Italy, including the authors of this article, were quarantined at the time we collected and analyzed the data, so we were forced to opt for an online methodology of data collection.

While methodologically limited, our results could open a number of possible future studies. First, this study could be considered as a time-zero data collection for a longitudinal study. In this regard, we would contact our previous participants in order to ask if they will participate to further data collection. Thus, we could follow the change in risk perception and psychological situation in the general population and healthcare workers during the evolution of COVID-19 infection spread. More experimental and cross-sectional studies are requested in order to better understand the relationship between healthcare workers’ and the general population’s information gap and risk perception in a pandemic disease scenario. This could help scientific community to find new strategies for conveying lifesaving information to population. Reducing such information gap could also help in reducing the sense of separation between the healthcare workers and the rest of population and thus the sense of isolation with its negative psychological consequences on both groups.

## Conclusion

Our study supports that a difference in risk perception between health workers and the general population exists and suggests a number of explanations for its causes as well as possible solutions to reduce it, with benefits in the psychological conditions of both groups of participants. More efforts need to be done in this direction, also because reducing psychological distress could advantage physical health state (Prince et al., 2007), in particular for medical staff facing such a difficult time, improving the quality of care they could provide (Maunder, 2009; Imai, 2020).

## Data Availability Statement

The raw data supporting the conclusions of this article will be made available by the authors, without undue reservation, to any qualified researcher.

## Ethics Statement

Ethical review and approval was not required for the study on human participants in accordance with the local legislation and institutional requirements. The patients/participants provided their written informed consent to participate in this study.

## Author Contributions

LS and CG designed the study and administered the questionnaire. LS conducted the data analysis. LS and CG wrote and revised the manuscript. Both authors contributed to the article and approved the submitted version.

## Conflict of Interest

The authors declare that the research was conducted in the absence of any commercial or financial relationships that could be construed as a potential conflict of interest.
