# Supplementary material for: Differences Between Health Workers and General Population in Risk Perception, Behaviors, and Psychological Distress Related to COVID-19 Spread in Italy
Source: Front Psychol. 2020 Sep 4;11:2166. doi: 10.3389/fpsyg.2020.02166 (PMC7499804; doi:10.3389/fpsyg.2020.02166)
Supplement: Supplementary file 1 [file Table_1.DOCX]

**Appendix A**

**Table A. COVID-19 Questionnaire items in Italian (as presented to participants) and in English translation.**

| **N°** | **Original items in Italian** | **English translation** |
| --- | --- | --- |
| 1 | Hai fatto il tampone per il SARS-CoV-2? | Have you done a throat swab for SARS-CoV-2? |
| 2 | Se sì, sei risultato positivo? (se hai risposto "no" alla domanda precedente, seleziona "no") | If yes, was it positive? (answer “no” if you have answered “no” at the previous question) |
| 3 | Hai o hai avuto di recente uno o più sintomi riferibili alla COVID-19 (febbre, stanchezza, tosse secca, indolenzimento, dolori muscolari, congestione nasale, naso che cola, mal di gola, diarrea, polmonite)? | Do you or have you recently had one or more symptoms related to COVID-19 (fever, fatigue, dry cough, soreness, muscle pain, nasal congestion, runny nose, sore throat, diarrhea, pneumonia)? |
| 4 | Se sì, pensi che possa trattarsi di COVID-19? (se hai risposto "no" alla domanda precedente, seleziona "no") | If yes, did you think could be COVID-19? (answer “no” if you have answered “no” at the previous question) |
| 5 | Se sì, hai allertato il servizio sanitario nazionale? (se hai risposto "no" alla domanda precedente, seleziona "no") | If yes, have you alerted the national health service? (answer “no” if you have answered “no” at the previous question) |
| 6 | Sei attualmente o sei stato in regime di quarantena spontanea o imposta a causa del COVID-19? | Are you currently or have you been on spontaneous or imposed quarantine due to COVID-19? |
| 7 | Sei attualmente o sei stato di recente a contatto con persone a forte rischio infettivo? | Are you currently or have you recently been in contact with people at high infectious risk? |
| 8 | Sei attualmente o sei stato a contatto di recente con persone risultate positive al tampone per COVID-19? | Are you currently or have you recently been in contact with people who had a positive test for COVID-19? |
| 9 | Nella tua zona o città, sono stati rilevati casi positivi di infezione da COVID-19? | Have any positive cases of COVID-19 infection been detected in your area or city? |
| 10 | Pensi di essere a rischio infettivo attualmente? | Do you think you are currently at infectious risk? |
| 11 | Pensavi di essere a rischio quando sono apparsi i primi casi in Italia a fine gennaio 2020? | Did you think you were at risk when the first cases appeared in Italy in January 2020? |
| 12 | Pensi che i tuoi familiari/cari siano a rischio infettivo attualmente? | Do you think your family members/loved ones are currently at infectious risk? |
| 13 | Sei preoccupato dalla possibilità che, in caso di infezione, potresti avere delle complicanze anche gravi o morire? | Are you worried about the possibility that, in case of infection, you may have serious complications or die? |
| 14 | Sei preoccupato dalla possibilità che, in caso di infezione, qualcuno dei tuoi familiari/cari possa avere delle complicanze anche gravi o morire? | Are you worried about the possibility that, in case of infection, some of your family/loved ones may have even serious complications or die? |
| 15 | Pensi che la diffusione del virus proseguirà agli attuali ritmi di contagio, rallenterà o accelererà nei prossimi giorni? | Do you think that the spread of the virus will continue at the current rate of contagion, will slow down or accelerate in the next few days? |
| 16 | Pensi che la diffusione del virus proseguirà agli attuali ritmi di contagio, rallenterà o accelererà nelle prossime settimane? | Do you think that virus spread will continue at the current rate of contagion, will slow down or accelerate in the next weeks? |
| 17 | Sei preoccupato dalla possibilità che la situazione possa precipitare a livello globale nel prossimo futuro a causa della COVID-19? | Are you worried about the possibility that the situation may precipitate at global level in the near future due to COVID-19? |
| 18 | Sei preoccupato dalla possibilità che, in caso il sistema sanitario nazionale non fosse in grado di garantire le cure o di sostenere il volume di pazienti ricoverati, possano accadere episodi di violenza e sopraffazione tra i pazienti o loro familiari? | Are you worried about the possibility that, if the national health system was unable to guarantee treatment or to support the volume of hospitalized patients, episodes of violence and abuse may occur among patients or their families? |
| 19 | Sei preoccupato dalla possibilità che il comportamento delle altre persone in risposta a questa situazione sia più pericoloso rispetto ai rischi medici associati all'infezione da COVID-19? | Are you concerned about the possibility that other people’s behavior in response to this situation could be more dangerous than the medical risks associated with COVID-19 infection? |
| 20 | Se lavori in ambito medico/sanitario, temi che la scarsità di mezzi e risorse di cura prevista per il prossimo futuro possa esporti ad episodi di violenza o ritorsione da parte di pazienti o dei loro familiari? (rispondi "no" se non lavori in ambito medico/sanitario) | If you work in the medical/health sector, do you fear that the scarcity of means and resources of care foreseen for the near future could expose you to episodes of violence or retaliation by patients or their families? (answer “no” if you are not a doctor/other health worker) |
| 21 | Qual è, attualmente, la tua preoccupazione maggiore rispetto al COVID-19? (se non sei preoccupato, scrivi "no") | At this moment, what is it your biggest concern about COVID-19? (if you’re not worried, write "no") |
| 22 | Pensi che tu possa aver messo a rischio di contagio te stesso con i tuoi comportamenti? | Do you think you might have put yourself at risk of infecting yourself with your behavior? |
| 23 | Pensi che tu possa aver messo a rischio di contagio i tuoi familiari/cari con i tuoi comportamenti? | Do you think you might have put yourself at risk of infecting your family/loved ones with your behavior? |
| 24 | Stai attuando le disposizioni igienico-sanitarie di prevenzione come lavarsi spesso le mani, evitare contatti fisici (strette di mano, baci e abbracci), igienizzare le superfici, tenersi a certa distanza dagli interlocutori? | Are you implementing the hygienic-sanitary prevention provisions such as washing your hands often, avoiding physical contacts (handshakes, kisses and hugs), sanitizing surfaces, keeping a certain distance from the interlocutors? |
| 25 | Pensi che le persone si stiano comportando in modo adeguato alla situazione? | Do you think people are having appropriate behaviors for the situation? |
| 26 | Sei preoccupato/arrabbiato rispetto alla violazione delle disposizioni di contenimento mostrata da alcune persone in questi ultimi giorni? | Are you worried/angry about the violation of containment provisions shown by some people in the last few days? |
| 27 | Pensi che sarebbe giusto punire con maggiore severità comportamenti rischiosi per la salute pubblica? | Do you think it would be right to punish more severely public health risky behaviors? |
| 28 | Continui attualmente o hai continuato negli ultimi giorni a frequentare luoghi di aggregazione a fini ricreativi come pub, ristoranti, centri commerciali, fiere, eventi, cinema o teatri? | Are you continuing or have you continued in the last few days to attend meeting places for recreational purposes such as pubs, restaurants, malls, fairs, events, cinemas or theatres? |
| 29 | Hai di recente comprato una quantità maggiore di cibo in scatola, a lunga conservazione e/o acqua in bottiglia per farne scorta? | Have you recently bought more canned, long-life food and/or bottled water to stock up on it? |
| 30 | In caso di diffusione del virus nella tua zona di residenza, cercheresti in ogni modo di spostarti verso una zona considerata più sicura? | If the virus spread in your living area, would you try in any way to move to an area considered safer? |
| 31 | Pensi che la preoccupazione e la paura relativa alla COVID-19 sia una motivazione valida per violare le disposizioni sanitarie di contenimento? | Do you think the concern and fear surrounding COVID-19 is a valid reason to violate the sanitary containment provisions? |
| 32 | Pensi che attualmente il sistema sanitario nazionale sarebbe in grado di prendersi cura di te se ti dovessi infettare? | Do you think the national health system would currently be able to take care of you if you got infected? |
| 33 | Pensi che sia giusto dare priorità di cura alle persone con maggiore speranza di sopravvivenza in caso di necessità o di carenza di posti letto in ospedale? | Do you think it is right to give care priority to people with greater hope of survival in case of need or shortage of hospital beds? |
| 34 | Se a te o a uno dei tuoi familiari/cari dovesse essere negato l'accesso a cure intensive per dare priorità a pazienti con maggiore probabilità di sopravvivenza, accetteresti questa decisione di buon grado? | If you or one of your family/loved ones were prevented from accessing to intensive care units in order to give priority to patients with a higher probability of survival, would you accept this decision at all kindly? |
| 35 | Pensi che sia opportuno cercare di accedere alle cure con qualunque mezzo, anche violento o illegale? | Do you think it is appropriate to try to have access to treatment by any means, including violent or illegal? |
| 36 | Pensi che l'azione di contenimento del virus sia necessaria? | Do you think that virus containment measures of the virus are necessary? |
| 37 | Pensi che l'azione di contenimento attualmente disposta sia adeguata? | Do you judge the current containment measures as adequate? |
| 38 | Pensi che le misure di contenimento debbano essere migliorate o rafforzate? | Do you think that the containment measures need to be improved or strengthened? |
| 39 | Pensi che sia giusto impiegare l'esercito o la forza pubblica per far rispettare le misure di contenimento sanitario? | Do you think it is right to use the army or the public force in order to enforce health containment measures? |
| 40 | Pensi che sia giusto limitare la libertà delle persone in vista di un maggior contenimento del virus? | Do you think it is right to limit people's freedom in view of a greater virus containment? |
| 41 | Pensi che sia giusto limitare autonomamente i propri comportamenti a rischio (ad esempio, evitare viaggi di piacere, non frequentare locali affollati, non partecipare ad eventi)? | Do you think it is right to limit your risky behaviors autonomously (for example, avoid leisure travel, do not attend crowded places, do not participate in events)? |
| 42 | Stai attualmente limitando i tuoi comportamenti a rischio? | Are you currently limiting your risky behavior? |
| 43 | Pensi di essere adeguatamente informato sulle caratteristiche della COVID-19? | Do you think you are properly informed about the characteristics of COVID-19? |
| 44 | Pensi di essere adeguatamente informato sulla situazione politico/sociale relativa alla COVID-19? | Do you think you are properly informed about the political/social situation related to COVID-19? |
| 45 | Pensi che ci sia bisogno di maggiore comunicazione da parte di esperti come virologi ed altri medici? | Do you think more communication from experts (such as virologists and other doctors) is needed? |
| 46 | Pensi che i media si occupino troppo o troppo insistentemente di COVID-19? | Do you think that media are too much or too insistently concerned with COVID-19? |
| 47 | Pensi che ci siano delle informazioni sensibili relative alla COVID-19 che ti sono nascoste? | Do you think there is any sensitive information, related to COVID-19, hidden from you? |
| 48 | Se sì, pensi che siano relative alla maggiore pericolosità reale del virus? (se hai risposto "no" alla domanda precedente, seleziona "no") | If yes, do you think they are related to a real greater danger of the virus? (if you answered "no" to the previous question, select "no") |
| 49 | Pensi che attualmente nell'opinione pubblica la percezione del rischio relativo alla COVID-19 sia maggiore, minore o adeguata rispetto a quanto dovrebbe essere? | Do you think that the perception of risk related to COVID-19 in public opinion is greater, lesser or adequate than it should be? |
